# Supplementary material for: Poly(ethylene glycol) Diacrylate Iongel Membranes Reinforced with Nanoclays for CO2 Separation
Source: Membranes (Basel). 2021 Dec 20;11(12):998. doi: 10.3390/membranes11120998 (PMC8703618; doi:10.3390/membranes11120998)
Supplement: Supplementary file 1 [file membranes-11-00998-s001.zip › membranes-1491334-supplementary.pdf]

membranes

*Supplementary Information*

# Poly(ethylene glycol) Diacrylate Iongel Membranes Reinforced with Nanoclays for $CO_2$ Separation

**Ana R. Nabais [1,†], Rute O. Francisco [1,†], Vítor D. Alves [2], Luísa A. Neves [1,*] and Liliana C. Tomé [1,*]**

[1] LAQV-REQUIMTE, Department of Chemistry, NOVA School of Science and Technology, FCT NOVA, Universidade Nova de Lisboa, 2829-516 Caparica, Portugal; a.nabais@campus.fct.unl.pt (A.R.N.); rr.francisco@campus.fct.unl.pt (R.O.F.)
[2] LEAF—Linking Landscape, Environment, Agriculture and Food—Research Center, Associated Laboratory TERRA, Instituto Superior de Agronomia, Universidade de Lisboa, Tapada da Ajuda, 1349-017 Lisabon, Portugal; vitoralves@isa.ulisboa.pt
* Correspondence: lan11892@fct.unl.pt (L.A.N.); liliana.tome@fct.unl.pt (L.C.T.)
† Ana R. Nabais and Rute O. Francisco contributed equally to this work.

## Scanning Electron Microscopy (SEM)

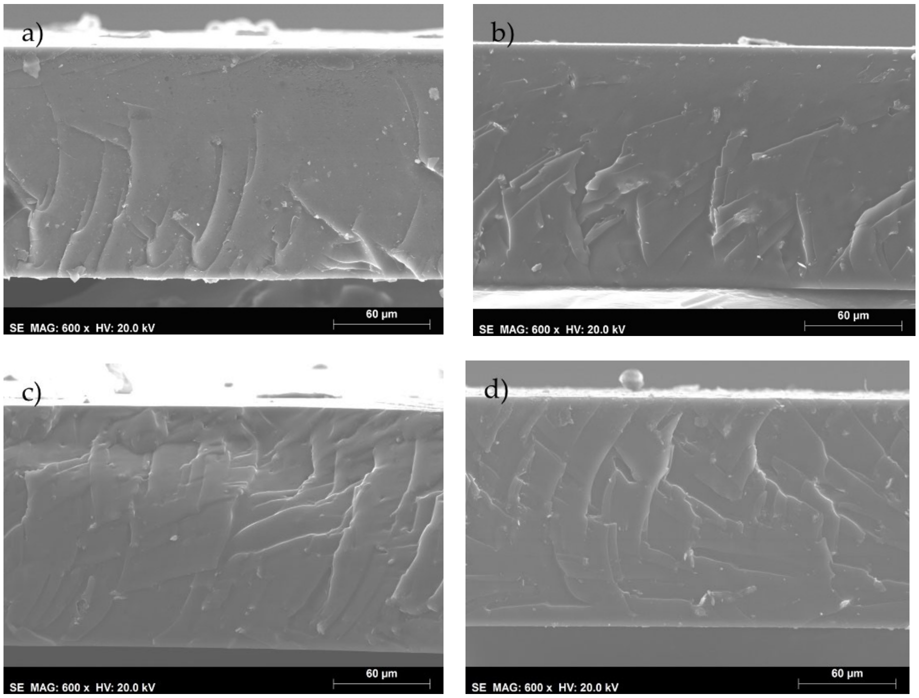


**Figure S1**. -SEM images of the iongels containing 0.5 **(a)**, 1 **(b)**, 2.5 **(c)** and 5 **(d)** wt% MMT.

## Attenuated Total Reflectance-Fourier Transform Infrared Spectroscopy (ATR-FTIR)

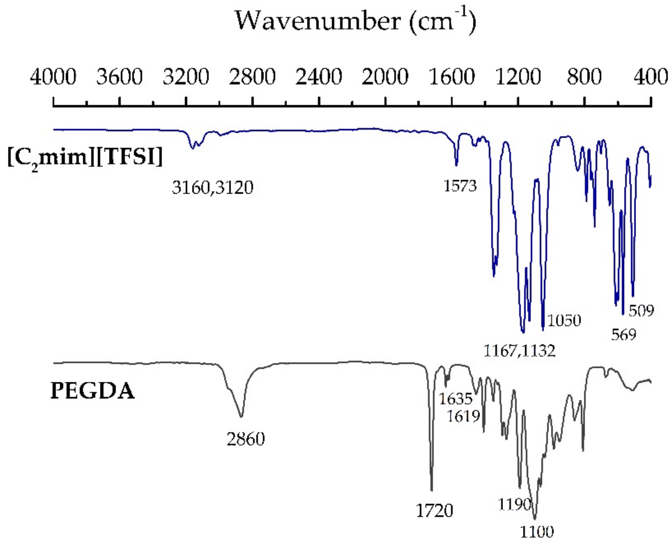


**Figure S2**. FTIR spectra of the [$C_2$mim][TFSI] IL and PEGDA network.

Thermogravimetric Analysis (TGA)

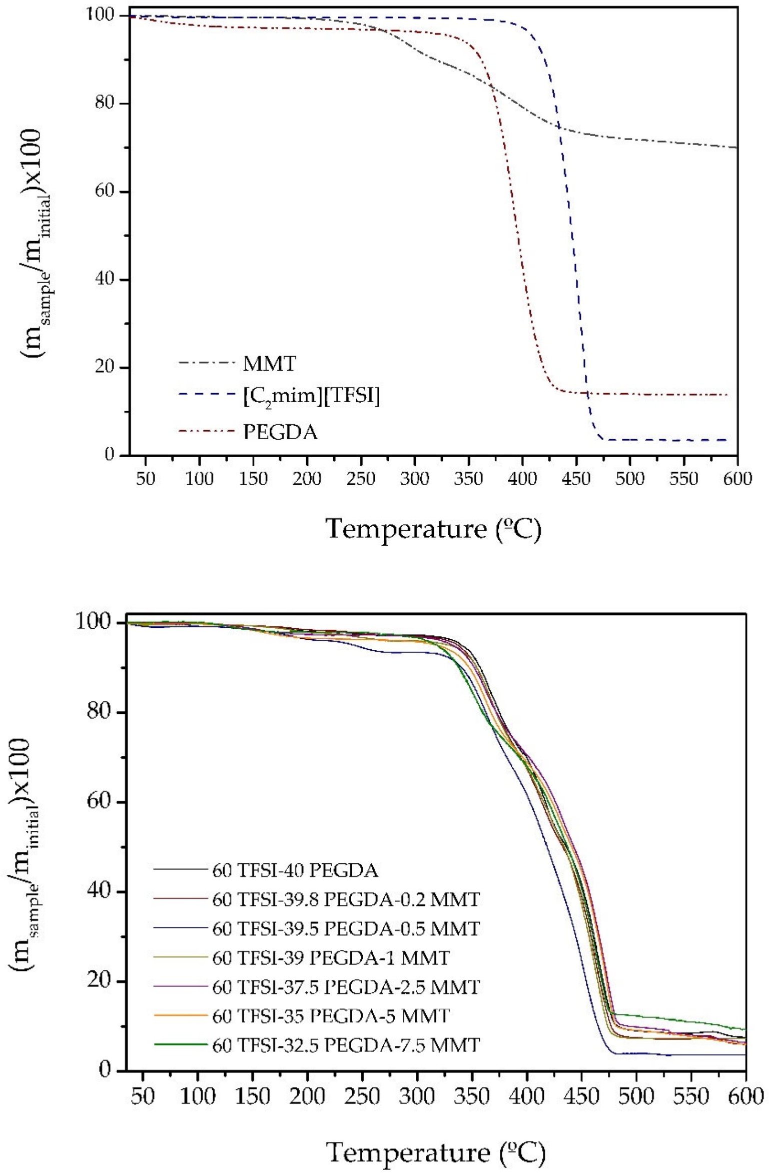


**Figure S3**. – Thermogravimetric profiles of the neat iongel components (top) and all iongels prepared with different MMT contents (bottom).

Gas permeation results

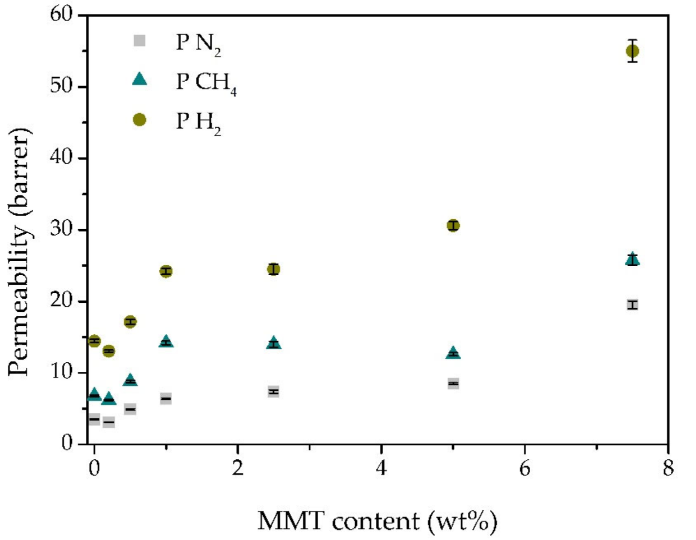


**Figure S4.** - $H_2$, $CH_4$ and $N_2$ permeabilities obtained for the prepared iongels (at 60 wt% IL), as a function of the MMT content.
